# Quantifying Spatial Genetic Structuring in Mesophotic Populations of the Precious Coral *Corallium rubrum*

**DOI:** 10.1371/journal.pone.0061546

**Published:** 2013-04-30

**Authors:** Federica Costantini, Lorenzo Carlesi, Marco Abbiati

**Affiliations:** 1 Dipartimento di Scienze Biologiche, Geologiche e Ambientali, Centro Interdipartimentale di Ricerca per le Scienze Ambientali, Università di Bologna, CoNISMa, Ravenna, Italy; 2 ISMAR CNR, Bologna, Italy; Heriot-Watt University, United Kingdom

## Abstract

While shallow water red coral populations have been overharvested in the past, nowadays, commercial harvesting shifted its pressure on mesophotic organisms. An understanding of red coral population structure, particularly larval dispersal patterns and connectivity among harvested populations is paramount to the viability of the species. In order to determine patterns of genetic spatial structuring of deep water *Corallium rubrum* populations, for the first time, colonies found between 58–118 m depth within the Tyrrhenian Sea were collected and analyzed. Ten microsatellite loci and two regions of mitochondrial DNA (mtMSH and mtC) were used to quantify patterns of genetic diversity within populations and to define population structuring at spatial scales from tens of metres to hundreds of kilometres. Microsatellites showed heterozygote deficiencies in all populations. Significant levels of genetic differentiation were observed at all investigated spatial scales, suggesting that populations are likely to be isolated. This differentiation may by the results of biological interactions, occurring within a small spatial scale and/or abiotic factors acting at a larger scale. Mitochondrial markers revealed significant genetic structuring at spatial scales greater then 100 km showing the occurrence of a barrier to gene flow between northern and southern Tyrrhenian populations. These findings provide support for the establishment of marine protected areas in the deep sea and off-shore reefs, in order to effectively maintain genetic diversity of mesophotic red coral populations.

## Introduction

*Corallium rubrum* (L.1758), also known as precious red coral, is a gorgonian endemic to the Mediterranean Sea and Eastern Atlantic Ocean occurring as a large numbers of small sized colonies above 50 m depth ([1], hereafter named shallow-water populations), or as scattered larger colonies below 50 m down to the deeper margin of its distribution ([2], hereafter named deep-water populations). *C. rubrum* has been regarded as a precious commodity throughout history and has endured millennia of exploitation within the Mediterranean [3]. Historically, red coral harvesting affected mainly shallow water populations that were easily accessed by traditional methods. Centuries of exploitation led to a reduction in both size and abundance of these colonies [4]. In addition to anthropogenic pressures, shallow waters populations have been impacted by natural threats, such as boring sponges [5], and mass mortality events resulting from temperature increases (e.g. [6]).

In an effort to protect these endangered populations, recommendations made on 2011 by the FAO-GFCM Scientific Committee were adopted by imposing a ban of red coral harvesting at depths shallower then 50 m [7]. Declining availability of colonies coupled with the enforcement of the aforementioned regulations pushed red coral fishermen to harvest deeper populations found within the mesophotic biogenic reefs (50–150 m depth; [3]). Provide sound background scientific knowledge on the functioning of red coral mesophotic populations as well as on their relationship with shallow ones become a priority for the implementation of effective management and conservation policies.

Mesophotic biogenic reefs have recently been discovered along most continental shelfs [8]; [9]; [10]. They are characterized by the presence of corals and associated assemblages, and show high species diversity compared to the surrounding sea bed [11]; [12]. Previous studies suggested that in the past mesophotic biogenic reefs may have acted as refugia (where species could survive during periods of adverse conditions elsewhere) and/or source of propagules for threatened shallow-water populations [11]; [13]; [14]; [15]; [16]. However, deep marine ecosystems are now affected by anthropogenic impacts, similar to those historically inflicted upon shallow water coastal habitats. These includes unsustainable harvesting practices, pollution, habitat loss and fragmentation, and a numerous threats arising from climate change, and ocean acidification [17]. Human pressures may alter species distributions, population dynamics, growth rates and genetic structuring of deep biogenic reefs, questioning their role as refugia. Unfortunately, studies addressing the effectiveness of deep Anthozoa populations to act as a source of propagules for shallow-water populations provided conflicting results [18]; [14]. Complex patterns of connectivity emerging from these studies stress the need to increase our understanding of larval processes in mesophotic red coral populations.

Previous genetic studies on shallow-water red coral populations revealed strong structuring at both large (e.g. hundreds of km) and small (<1 m) scales [19], [20]; [21], [22]. Recently, a reduction of genetic variability in red coral populations along a depth gradient has been reported [23], suggesting that depth can affect patterns of genetic structuring in the species. Moreover, a threshold in connectivity was observed among samples collected across 40–50 m depth, supporting the occurrence of hidden barriers to larval dispersal separating adjacent zones (e.g. thermocline, water circulation) that could determine a genetic separation between shallow and mesophotic red coral populations [2], [23].

Knowledge concerning the biological and ecological features of red coral mesophotic populations, is clearly limited by the difficulties associated with accessing and obtaining samples from deep water habitats [24]; [23]. The use of Remotely Operated Vehicles (ROVs) facilitates exploration and surveying of the deep sea, including mesophotic reefs [8]; [9]; [25]; [26]. In this study for the first time samples of *Corallium rubrum* have been collected from mesophotic habitats (58–118 m depth) to investigate patterns of spatial genetic structuring. Ten microsatellite loci and two portions of the mitochondrial DNA (mutS homolog gene and mtC putative control region) were used to analyse the collected specimens. The main goals of the present study were to quantify patterns of genetic diversity in mesophotic red coral populations, and to define population structuring at different spatial scales (from tens of metres to hundreds of kilometres). Implications of these new findings for conservation and management of mesophotic red coral populations are discussed.

## Materials and Methods

### Sample Collection

In July 2010 an oceanographic cruise by the RV Astrea was dedicated to the survey and sampling of mesophotic red coral populations thanks to the project “Study of red coral deep dwelling populations: structure, demography and genetics" supported and financed by the Italian Ministry of Environment. No specific permissions were required for sampling in Campania area, while for sampling in Tuscany area we obtained permission for field activity thanks to the Director of the Arcipelago Toscano National Park.

**Figure 1:**
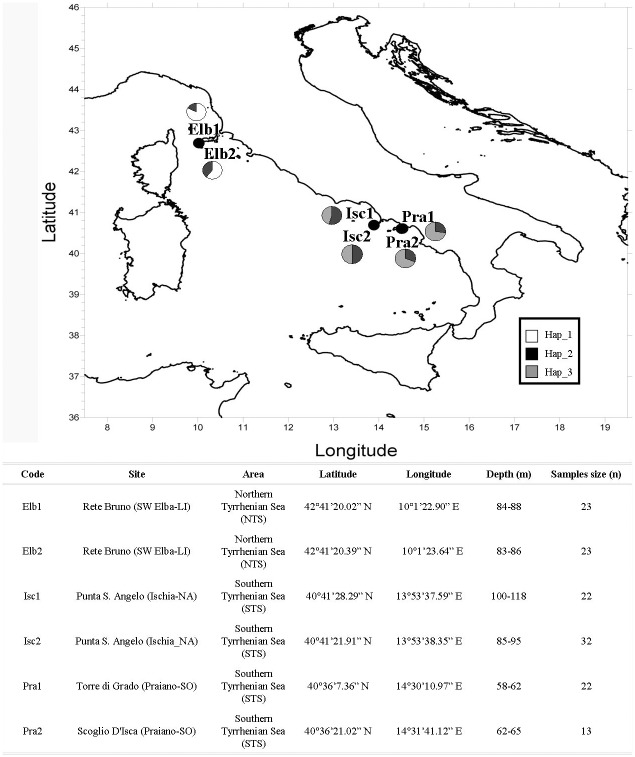
Map of the sampling sites, including a table with identification codes, sites, areas, geographical coordinates, depths and sample size. Pie charts represent mtC haplotype frequencies for each population.

Commercial SCUBA divers and a Remotely Operated Vehicle (ROV) collected 143 red coral colonies in two areas at depth ranging between 58 and 118 metres. The investigated areas were the Northern Tyrrhenian Sea (NTS) and the Southern Tyrrhenian Sea (STS), geographically separated by hundreds of kilometres (Fig. 1). Potential sampling sites were identified following previous accounts of deep-water red coral colonies made by professional fishermen. In the NTS area two populations were sampled southwest of Elba Island (Elb1, Elb2). In the STS, two populations from Ischia island (Isc1, Isc2) and two off Praiano (Pra1, Pra2) were sampled. Within each area populations are separated by tens of metres to tens of kilometres (see Table in Fig. 1).

For each population, fragments of branches were collected from 15 to 33 live red coral colonies. Sampling was done within an area of about 50–100 m^2^. Fragments were preserved in 80% ethanol and stored at 4°C. Total genomic DNA was extracted using cetyltrimethyl ammonium bromide (CTAB) protocol.

Ten microsatellites specifically developed for *C. rubrum,* COR9, COR15, COR46, COR48, COR58 [27] and MIC20, MIC22, MIC23, MIC24, MIC26 [22] were analysed. Microsatellites were amplified either locus by locus, or in multiplex using a QIAGEN® Multiplex PCR Kit (see Table 1 for PCR multiplex set) using polymerase chain reaction (PCR) conditions described in [23].

**Table 1:** Summary of genetic diversity at eight microsatellite loci within *Corallium rubrum* populations.

LOCUS		POPULATIONS (n)						MEANS
		Elb1 (23)	Elb2 (24)	Isc1 (22)	Isc2 (33)	Pra1 (26)	Pra2 (15)	
**COR9^a^**								
N		21	19	22	31	22	13	21.33
Ar		7.22	7.31	3.00	3.87	5.65	5.84	5.48
H_O_		0.10	0.58	0.14	0.16	0.09	0.08	0.19
H_S_		0.77	0.81	0.58	0.70	0.68	0.50	0.67
F_IS_		**0.88**	**0.31**	**0.77**	**0.78**	**0.87**	**0.86**	0.74
**COR15^b^**								
N		22	23	22	31	21	13	22.00
Ar		2.80	2.00	2.71	2.02	2.57	1.92	2.34
H_O_		0.00	0.00	0.09	0.10	0.05	0.08	0.05
H_S_		0.42	0.39	0.21	0.09	0.32	0.07	0.25
F_IS_		**1.00**	**1.00**	**0.58**	−0.02	**0.86**	0.00	0.57
**COR46^a^**								
N		19	23	20	31	22	13	21.33
Ar		6.81	6.52	4.60	7.45	6.98	9.69	7.01
H_O_		0.58	0.43	0.55	0.61	0.95	0.85	0.66
H_S_		0.82	0.78	0.74	0.79	0.80	0.87	0.80
F_IS_		**0.32**	**0.46**	0.28	0.24	−0.16	0.06	0.20
**COR48^b^**								
N		21	23	22	23	21	12	20.33
Ar		9.38	5.90	5.26	8.58	9.63	9.00	7.96
H_O_		0.71	0.43	0.32	0.30	0.57	0.58	0.49
H_S_		0.88	0.81	0.74	0.86	0.87	0.85	0.84
F_IS_		**0.21**	**0.48**	**0.59**	**0.66**	**0.37**	**0.36**	0.44
**COR58^a^**								
N		21	20	20	32	22	13	21.33
Ar		5.39	2.94	9.73	9.79	7.99	6.84	7.11
H_O_		0.52	0.40	0.60	0.50	0.59	0.62	0.54
H_S_		0.75	0.45	0.88	0.85	0.82	0.74	0.75
F_IS_		**0.32**	0.14	**0.34**	**0.42**	**0.30**	**0.21**	0.29
**MIC20^c^**								
N		23	23	22	32	22	13	22.50
Ar		6.45	5.76	4.92	7.12	8.74	5.92	6.48
H_O_		0.43	0.39	0.55	0.59	0.86	0.77	0.60
H_S_		0.64	0.65	0.57	0.77	0.80	0.76	0.70
F_IS_		**0.34**	**0.42**	0.07	0.25	−0.05	0.03	0.18
**MIC24^c^**								
N		23	23	22	32	22	13	22.50
Ar		5.03	4.82	7.64	6.35	4.27	3.77	5.31
H_O_		0.30	0.48	0.64	0.47	0.27	0.23	0.40
H_S_		0.66	0.68	0.75	0.69	0.25	0.21	0.54
F_IS_		**0.56**	**0.32**	0.17	**0.34**	−0.06	−0.04	0.21
**MIC26^c^**								
N		22	23	22	32	22	13	22.33
Ar		10.49	8.37	9.02	10.42	10.14	7.84	9.38
H_O_		0.95	0.87	0.77	0.72	0.91	0.77	0.83
H_S_		0.86	0.84	0.88	0.89	0.88	0.83	0.86
F_IS_		−**0.08**	−0.02	0.14	**0.21**	−0.01	0.11	0.06
**Multilocus**								
MLG	23		23	22	32	22	13	-
Ar	6.70		5.45	5.86	6.95	7.00	6.35	6.38
H_O_	0.45		0.45	0.46	0.43	0.54	0.50	0.47
H_S_	0.73		0.68	0.67	0.71	0.68	0.60	0.68
F_IS_	**0.40**		**0.36**	**0.34**	**0.40**	**0.23**	**0.22**	0.32

n, number of sampled colonies; N, number of genotypes per locus; Ar, allelic richness based on 12 individuals; H_o_, observed heterozygosity; H_s_, gene diversity ( [35]); *F_IS_*, [39] estimate of Wright's (1951) [67] fixation index; MLG, multilocus genotypes per sample. Bold types indicate significant deviations from HWE after FDR correction. ^a^, loci amplified in multiplex PCR 1; ^b^, loci amplified in multiplex PCR 2; ^c^, loci amplified in multiplex PCR 3.

Genotyping of individuals was carried out on an ABI 310 Genetic Analyser (Applied Biosystems), using forward primers labelled with FAM, HEX/VIC, TAMRA/NED, ROX/PET (Sigma) and LIZ HD500 (Applied Biosystems) as internal size standards. Allele sizing was conducted using GENESCAN Analysis Software version 2.02 (Applied Biosystems).

Amplification of mtMSH sequences was conducted using the primers MUT4759f and MSH5376r and following [28] and [29] protocols.

PCR amplifications of the mtC gene were performed using the primers ND618510CkonojF (5′-CCATAAAACTAGCTCCAACTATTCC-3′) and COI16CkonojR (5′-GGTTAGTAGAAAATAGCCAACGTG-3′) (Sigma) specifically developed in this study. These primers were designed using the online PRIMER3 version 4.0 software [30] on the nad6 and cox1 genes flanking the putative control region, likely to be located in the intergenic spacer 12 (IGS12) of the mitochondrial genome of *Paracorallium japonicum* and *Corallium konojoi* [31]. Each 12.5 µL PCR reaction contained approximately 20 ng DNA, 1X PCR buffer (Invitrogen), 2 mM MgCl2, 0.5 µM of each primer, 0.8 mM dNTPs and 1 U Taq polymerase (Invitrogen). Amplifications were performed on a GeneAMP PCR System 2700 (Applied Biosystems) as follows: an initial denaturation at 95°C for 3 min, 30 cycles including 95°C for 30 s, 59°C for 30 s, 72°C for 60 s, followed by a final extension at 72°C for 5 min. PCR products were sent to Macrogen (South Korea) for purification and sequencing, while the obtained sequences were edited and aligned manually using MEGA version 5.0 [32]. The mtC haplotypes generated in this study were lodged with GenBank (Accession N. KC597700-KC597702).

### Genetic Variability

Sampling using ROVs may cause a fragmentation of the colonies; and so little branches from the same colonies could be sampled. To avoid this problem fragments sharing the same multilocus genotype (MLG) were checked using GENALEX version 6.1 [33]. The unbiased probability of identity (P_ID_ [34]) that two individuals share the same MLG by chance and not by descent was computed. Microsatellite diversity within populations for each locus and over all loci was estimated as observed heterozygosity (*H_O_*) and unbiased gene diversity (*H_S_*, [35]) using the GENETIX software package version 4.05 [36]. As the number of alleles found in a sample is dependent on sample size, allelic richness (Ar) was estimated using the [37] rarefaction index in FSTAT version 2.9.3.2 ( [38]) with a sample size of 12 specimens.

Single and multilocus *F_I_*_S_ were estimated using Weir and Cockerham’s *f* [39] and significant departures from the Hardy–Weinberg equilibrium (HWE) were tested using the exact test implemented in GENEPOP version 4.1 [40], with the level of significance determined by Markov-chain randomization (1000 dememorizations, 100 batches and 1000 iterations per batch).

Populations were grouped according to their geographical origin (A: three groups: NTS-Elba, STS-Ischia and STS-Praiano; and B: two groups: NTS and STS). Significant differences in genetic diversity (*H_O_, H_S_* and Ar) among groups of populations were tested using a permutation procedure (1000 iterations) in FSTAT.

Sequence genetic diversity within samples was estimated using haplotype diversity (h, [35]) and nucleotide diversity (π, [35]) executed via ARLEQUIN version 3.5 software [41].

### Population Structure Analysis

Due to deviations from the Hardy–Weinberg equilibrium, genotypic differentiation between populations was tested with an exact test (Markov chain parameters: 1000 dememorizations, followed by 100 batches of 1000 iterations per batch) using GENEPOP. As null alleles can induce overestimation of genetic distance, pairwise *F_ST_* estimates were computed following the excluding null alleles (ENA) method in FREENA [42]. The significance of pairwise genotyping differentiation between populations was tested with an exact test using GENEPOP and Jost’s actual measure of differentiation, *D_est_* [43], calculated using DEMEtics version 0.8.3 [44] within the statistical package R v2.13.1 [45]. Overall estimates of *D_est_* were calculated from individual loci using a harmonic mean approximation. P-values were obtained via bootstrap methods with 1000 pseudo-replications.

For the mtMSH and mtC sequence data sets, genetic differentiation between populations was estimated using pairwise *F_ST_* estimator and its significance was determined using a permutation test (10000 permutations) in ARLEQUIN.

For both microsatellite and sequence data sets, models of isolation by distance between populations were tested through a Mantel test [46] computed using the Isolde program implemented in GENEPOP. A significant correlation between genetic differentiation estimates (*F_ST_* and *D_est_*) and the geographical distances among populations were tested using 1000 permutations.

The partition of the genetic variance among populations based on the three data sets was conducted using an analysis of molecular variance (AMOVA) in ARLEQUIN. With this purpose, red coral populations were grouped according to (A) sampling site (three groups: Elba, Ischia and Praiano) and (B) area (two groups: NTS and STS).

Discriminant Analysis of Principal Components (DAPC) [47], a multivariate model-free method, was performed to infer population subdivision. A file including microsatellite and mtDNA data was used as input. DAPC is a multivariate analysis that integrates principal component analysis (PCA) with discriminant analysis to summarize genetic differentiation between groups. It was implemented with the ADEGENET package [48] within the statistical package R version 2.12.1 in which 40 principal components of the PCA were retained, accounting for approximately 85% of the total genetic variability.

Assessment of structure within studied genotypes without a priori assumptions regarding populations was performed using a Bayesian clustering analysis implemented in STRUCTURE v2.3 [49]. The number of genetically homologous groups (K) was determined using the ad hoc statistic ΔK based on the rate of change in the log likelihood of data between consecutive K values as recommended by Evanno [50]. Mean and variance of log likelihoods of the number of clusters for K  = 1 to K  = 8 were inferred from multilocus genotypes by running structure five times with 500 000 repetitions each (burn-in  = 50 000 iterations) under the admixture ancestry model and the assumption of correlated allele frequencies among samples as suggested in [51].

When necessary, significance levels were corrected using a false discovery rate (FDR) methodology [52].

## Results

### Microsatellite Loci Variability

Six shared multilocus genotypes (MLGs) between colonies were found. One MLG was encountered twice in Elb2, two MLGs in Pra2 and one MLG in Isc2. Within Pra1 two MLGs were encountered twice and another was encountered three times. The probability that each of these genotypes was produced through sexual reproduction was low (P_ID_  = 1.16×10^−11^). Moreover, specimens sharing the same MLG showed identical mtMSH and mtC sequences. Both these results suggest that shared genotypes derive from fragmentation of single individual colonies, and therefore were included only once, obtaining a final data set of 135 different multilocus genotypes (Table 1).

Loci MIC22 and MIC23 were excluded from subsequent analysis as they amplified only in NTS populations (Elb1, Elb2) but did not give any amplification in STS populations (Isc1, Isc2 and Pra1, Pra2), even though repeated attempts were made. Lack of amplification may be caused by the occurrence of mutations in the microsatellites flanking region. These results deserve careful evaluation since they may disclose occurrence of a major barrier to gene flow between STS and NTS areas.

The eight microsatellites analysed were polymorphic in all samples. Throughout all samples, the number of alleles per locus ranged from 6 to 22, and allelic richness from 2.34 to 9.38 (Table 1). Within samples the allelic richness based on a minimum sample size of 12 diploid individuals ranged between 5.45 and 7. Mean observed heterozygosity ranged between 0.43±0.08 (in Isc2) and 0.54±0.13 (in Pra1), while gene diversity ranged from 0.60±0.11 (in Pra2) to 0.73±0.05 (in Elb1). Highly significant deviations from HWE were observed in all samples.

Multilocus estimates of *F_IS_* ranged from 0.22 to 0.4, showing heterozygote deficiencies in all analyzed samples (Table 1).

All the considered indexes of genetic variability showed similar ranges among populations (grouped by sampling sites – Elba, Ischia and Praiano; and by areas – NTS and STS). Indeed, observed heterozygosity, gene diversity, allelic richness and *F_IS_* did not significantly differ among groups (*P*>0.05).

### Mitochondrial Sequence Variation

Across all 135 analysed colonies the mtMSH fragment was 567 bp in length. Sequences alignments showed the presence of two previously recorded haplotypes (GenBank numbers GQ304902, GQ304903). Haplotype GQ304903 was present in all populations and was the most common (94%). Haplotype GQ304902 occurred only in Praiano populations, with six individuals in Pra1 and two individuals in Pra2, showing a haplotype diversity of 0.42 and 0.28, respectively (Table 2).

**Table 2:** Differences in sequences, distribution and genetic diversity of the two mtMSH and three mtC haplotypes found in *Corallium rubrum* populations.

	Nucleotide position				mtMSH					
	156				Elb1	Elb2	Isc1	Isc2	Pra1	Pra2
GQ304903	G				23	23	22	32	16	11
GQ304902	A								6	2
H					1	1	1	1	2	2
*h*					0	0	0	0	0.42	0.28
π					0	0	0	0	0.0007	0.0005
	**Nucleotide position**				**MtC**					
	**28**	**82**	**274**	**275**	**Elb1**	**Elb2**	**Isc1**	**Isc2**	**Pra1**	**Pra2**
Hap_1	C	A	A	G	19	14				
Hap_2	T	G	.	.	4	9	12	16	6	4
Hap_3	.	.	C	T			10	16	16	9
H					2	2	2	2	2	2
*h*					0.3	0.5	0.52	0.52	0.42	0.46
π					0.0021	0.0035	0.0036	0.0036	0.0029	0.0032

Dots indicate identical bases; H, total number of haplotypes; *h*, haplotype diversity (*h*, [35]); *π*, nucleotide diversity (*π*, Nei 1987 [35]). Accession number Hap_1: KC597700; Hap_2: KC597701; Hap_3: KC597701.

In this study the putative control region (mtC) has been analysed for the first time in *C. rubrum*, revealing a length of 290 bp, corresponding to positions 18624–18913 of the mitochondrial genome sequence of *Paracorallium japonicum* (GenBank number AB595189) and to positions 18565–18702 and 18815–18969 of the mitochondrial genome sequence of *Corallium konojoi* (GenBank number AB595190). The alignment of all the sequences showed the presence of four nucleotide substitutions (1.4% variation), identifying three different haplotypes (Table 2). Both areas showed a private haplotypes, Hap_1 and Hap_3 for NTS and STS, respectively. Hap_2 was present in all populations; nevertheless, Hap2 and Hap3 showed similar abundances (37.8%). Hap_1 in Elba populations was much more abundant compared to Hap_2 (72% and 28%, respectively). Low and comparable values of haplotype and nucleotide diversity of mtC were found among populations, with mean values of 0.45 (±0.03) and 0.0031 (±0.0002), respectively.

### Genetic Differentiation between Populations

**Figure 2:**
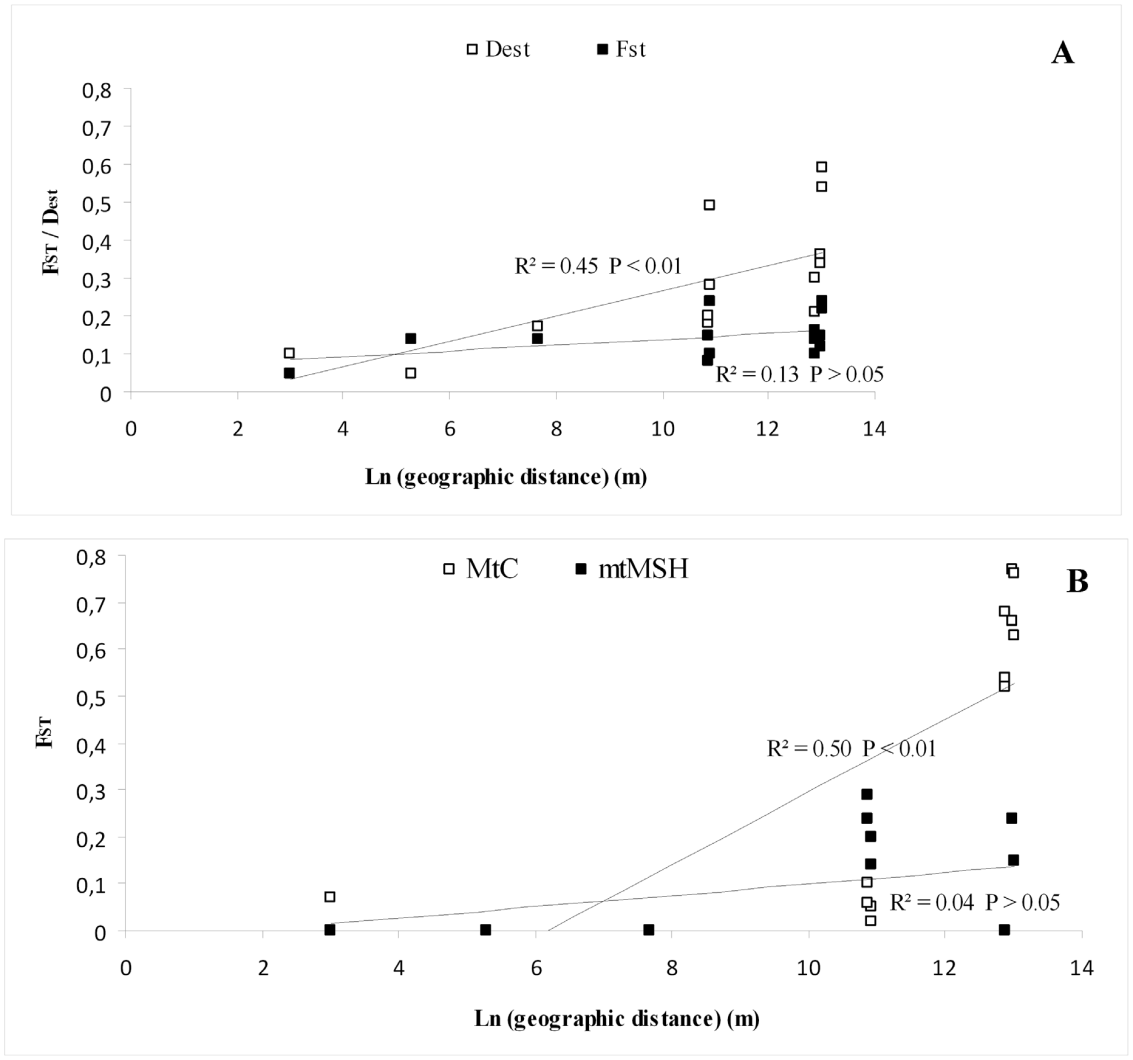
Relationship between genetic differentiation estimates and the logarithm of geographical distance among *Corallium rubrum* populations. A) relationship between genetic differentiation estimates (*F_ST_* and *D_est_*) and the logarithm of geographical distance for microsatellites markers. B) relationship between genetic differentiation estimates and the logarithm of geographical distance for both mtMSH and mtC markers.

Mitochondrial and nuclear molecular markers differ in their evolutionary rate and provide different estimates of the levels of polymorphism. A multi-marker approach is therefore recommended to provide accurate estimates of variability and differentiation among populations. The three molecular markers used in this study provided different estimates of genetic differentiation among populations. *F_ST_* estimates, as well as ENA *F_ST_* estimates, of the microsatellite dataset gave similar results. The pairwise *F_ST_* values ranged from 0.05 (Elb1 vs. Elb2) to 0.24 (Pra2 vs. Elb2 and Isc1) and all pairwise comparisons were significant after FDR correction (P<0.01; Table 3). *D_est_* values ranged from 0.05 (Isc1 vs. Isc2) to 0.59 (Elb2 vs. Pra2) and all pairwise comparisons were significant after FDR correction (*P*<0.01; Table 3). Overall, higher values of genetic differentiation were observed using *D_est_*, rather than *F_ST_* (average values *D_est_*  = 0.37; *F_ST_*  = 0.13). Based upon both *F_ST_* estimates and *D_est_*, Pra2 appeared to be the most differentiated of the populations. Moreover, the lowest values of *F_ST_* and *D_est_* were observed between populations belonging to the same area (Table 3). No correlation between *F_ST_* and the natural logarithm of the geographical distances was observed (*P*  = 0.08), whereas a significant isolation by distance pattern was detected using *D_est_* (*P*<0.01; Fig. 2A).

**Table 3:** Pairwise multilocus estimates of *F_ST_* below the diagonal, and *D_est_* above the diagonal between all *Corallium rubrum* populations.

	Elb1	Elb2	Isc1	Isc2	Pra1	Pra2
Elb1		**0.10**	**0.21**	**0.30**	**0.34**	**0.54**
Elb2	**0.05**		**0.30**	**0.30**	**0.36**	**0.59**
Isc1	**0.10**	**0.14**		**0.05**	**0.18**	**0.49**
Isc2	**0.15**	**0.16**	**0.14**		**0.20**	**0.28**
Pra1	**0.12**	**0.15**	**0.08**	**0.15**		**0.17**
Pra2	**0.22**	**0.24**	**0.24**	**0.10**	**0.14**	

All pairwise comparisons are statistically significant (*P*<0.01) after FDR correction.

The mtMSH marker provided the lowest values of genetic differentiation between samples. Pairwise *F_ST_* estimates ranged from −0.02 (Pra1 vs. Pra2) to 0.29 (Pra1 vs. Isc2) and all pairwise comparisons were not significant after FDR correction (Table 4). The newly developed mtC marker revealed intermediate values of genetic differentiation, compared to microsatellites and mtMSH. mtC pairwise *F_ST_* estimates ranged from 0 (Pra1 vs. Pra2 and Isc1 vs. Isc2) to 0.77 (Pra1 vs. Elb1). Global estimates of *F_ST_* were not significant both within NTS and STS areas (*F_ST_*  = 0.07, *P*>0.01; *F_ST_*  = 0.02, *P*>0.01, respectively). Pairwise multilocus estimates of *F_ST_* between NTS and STS populations were generally high (0.52–0.77) and significantly different from zero after FDR correction (Table 4).

**Table 4:** Pairwise multilocus estimates of both mtC *F_ST_* (below the diagonal) and mtMSH *F_ST_* (above the diagonal) between all *Corallium rubrum* populations.

	Elb1	Elb2	Isc1	Isc2	Pra1	Pra2
Elb1		0.00	0.00	0.00	0.24	0.15
Elb2	0.07		0.00	0.00	0.24	0.15
Isc1	**0.68**	**0.52**		0.00	0.24	0.14
Isc2	**0.68**	**0.54**	0		0.29	0.20
Pra1	**0.77**	**0.66**	0.10	0.07		0
Pra2	**0.76**	**0.63**	0.05	0.02	0	

Bold types indicate statistically significant values (*P*<0.01) after FDR corrections.

Significant isolation by distance was observed using *F_ST_* based on the mtC data set (*P*  = 0.01) (Fig. 2B), but not using *F_ST_* based on the mtMSH data set (*P*  = 0.12).

Patterns of variation among populations detected by AMOVA differed accordingly to the molecular makers and the groupings of the populations (comparing the 3 sampling sites versus the 2 areas) (Table 5). All the data sets showed a significant genetic variation within populations whatever the grouping (P<0.001) with a higher variation for the microsatellites. The greater variance among groups was observed for the mtC data set both between areas (NTS and STS; 56.78%) and among the sampling sites (Elba, Ischia, Praiano; 63.60%). The AMOVA on the mtMSH dataset showed that 23.65% of the variation was due to difference among the sampling sites.

**Table 5:** Analysis of molecular variance (AMOVA) among *Corallium rubrum* populations using microsatellites, mtMSH and MtC data sets.

		Microsatellites			mtMSH			MtC		
Source of variation		d.f	Variance components	%	d.f	Variance components	%	d.f	Variance components	%
*(A)*	*Sampling sites*									
	Among groups	2	0.132	4.34	2	0.014	23.65	2	0.594	56.78
	Among samples within groups	3	0.320	10.49**	3	0.000	−0.64**	3	−0.003	−0.30
	Within samples	264	2.597	85.17**	129	0.047	76.99**	129	0.455	43.52**
*(B)*	*Areas*									
	Among groups	1	0.153	4.96	1	−0.001	−2.27	1	0.828	63.60
	Among samples within groups	4	0.342	11.05**	4	0.012	20.58*	4	0.019	1.44
	Within samples	264	2.597	83.99**	129	0.050	81.69**	129	0.455	34.96**

Red coral populations were grouped according to (A) their sampling site (three groups: Elba, Ischia and Praiano) and (B) the areas (two groups: NTS and STS). ^*^ *P*<0.05, ^**^ *P*<0.001.

**Figure 3:**
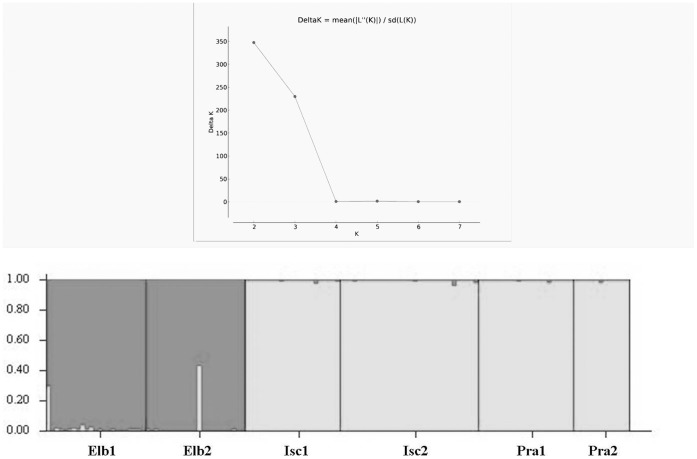
Above, values of ΔK, calculated as in [**50**] and based on the log likelihood of the data given by STRUCTURE for each number of clusters assumed (K). Below, results of the clustering analysis. In the bar plot, each of the individuals is represented by a vertical bar indicating its estimated proportion of membership to each cluster (represented by different colours).

**Figure 4:**
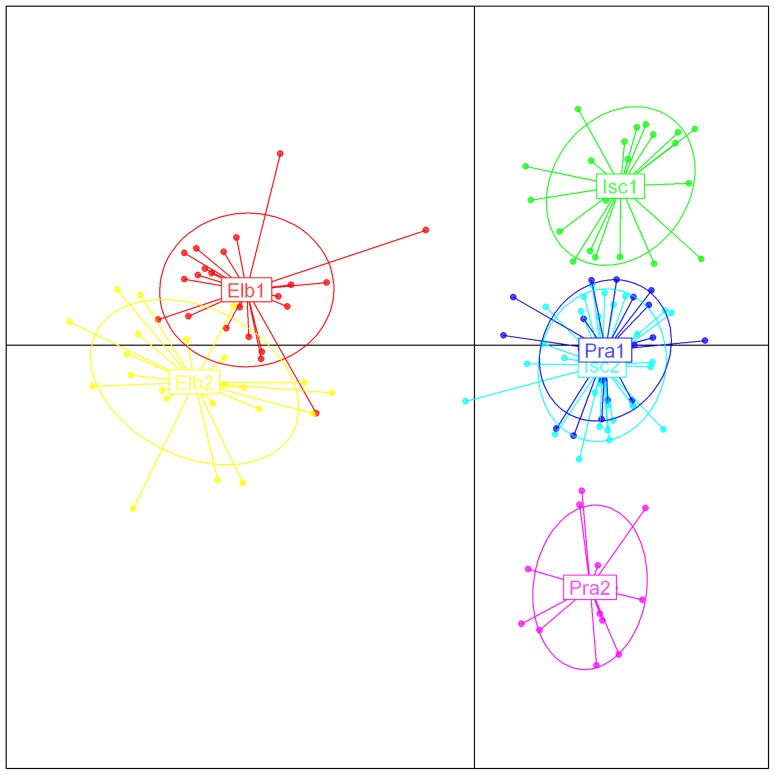
Subdivision of the red coral populations according to the DAPC method. Sampled populations are indicated with different colors, dots represent individual colonies.

Testing the significance of the stepwise clustering procedure done in STRUCTURE resulted in a separation of the populations into two clusters (cluster 1: Elb1 and Elb2; cluster 2: Pra1, Pra2, Isc1, Isc2; ΔK  = 347.56, Fig. 3). The DAPC on all populations (Fig. 4) revealed a clear separation between NTS and STS along the first axis, while the second axis shows the same degree of separation between populations within the sampling sites not detected by the STRUCTURE analysis.

## Discussion

This study provides the first data on genetic structuring in mesophotic *Corallium rubrum* populations which show genetic structuring at all the investigated scales (tens of metres to hundreds kilometres). A major barrier to gene flow has been revealed between the Northern Tyrrhenian and Southern Tyrrhenian Seas.

### Genetic Variability

Estimates of multilocus polymorphisms found in *Corallium rubrum* using microsatellites (mean H_S_  = 0.68±0.02) were low and comparable with those observed in populations at 50–70 metres depth by Costantini et al. [23] (Student’s *t*-test: *P*>0.05). A lack of significant differences in genetic variability between the NTS and STS, suggests that the low genetic variability of microsatellite loci is common to all mesophotic red coral populations. Diversity in population structure and dynamics between shallow and deep water red coral populations, as hypothesized by Bramanti et al. [53] and Linares et al. [4], may provide an explanation to differences in genetic variability. A multidisciplinary study comparing genetics, morphology, population structure and dynamics of mesophotic and shallow water red coral populations may help to reveal the relationship between biological characteristics and habitat features.

Strong deviations from Hardy-Weinberg equilibrium were detected in all populations and at all microsatellites. Depressed heterozygosity was already found in previous studies on *C. rubrum* [19], [20]; [21], [22], and confirms the occurrence in this species of processes affecting intra-population gene flow (see [20] for a detailed discussion). Microsatellites MIC22 and MIC23 amplified exclusively in the Northern Tyrrhenian Sea populations. Similar results were published by Aurelle et al. [54], who found that loci MIC22 and MIC23 were not amplified in Adriatic, Algerian and Alboran Sea populations, while they normally amplified in all populations from the north-western Mediterranean Sea. The absence of amplification in all red coral populations found south of the hypothetical line connecting Elba – Corsica – Sardinia - Balearic Islands suggests the lack of connectivity between populations on either side of this boundary. These results are in agreement with Bianchi & Morri [55], who hypothesised that this boundary could be a major Mediterranean biogeographic barrier. Occurrence in the Mediterranean sea of Northern-Southern biogeographic boundary is also supported by the results of the mitochondrial markers. In our study, we found the mtMSH “GQ304902” haplotype exclusively in the Southern site (i.e. Praiano populations). The mtMSH GQ304902 haplotype was previously found only in red coral populations sampled in the Southern Mediterranean Sea at a depth ranging between 600 and 800 metres [2]. Moreover, the Northern and Southern Tyrrhenian populations shared one common mitochondrial control region (mtC) haplotype, but they each possessed also a private haplotype.

In this study the mitochondrial control region (mtC) gene was for the first time amplified in red coral, and showed a higher variability compared to previously analysed mitochondrial genes (COI, mtMSH; [56]; [57]) which usually in Anthozoa have a low mutation rate. In fact, the mtC gene was already used by different authors to investigate connectivity among tropical and deep sea corals [13]; [58]; [59].

### Genetic Structuring

Our microsatellite dataset revealed that both the estimators of population divergence (*F_ST_* and *D_est_*) and AMOVA provide similar results. In fact, a significant genetic differentiation among *C. rubrum* populations at all the investigated spatial scales was detected, suggesting that mesophotic red coral populations are genetically structured. Small scale patterns of genetic structuring have been previously reported in shallow-water red coral populations [20]; [21]. Differentiation observed among populations could be explained by biological and ecological features, acting mainly at small spatial scales, and/or abiotic factors acting at larger scales. Red coral larvae have negative photo- and geo-taxis [60] and a low dispersal capability [61]; both of which could favour localized larval retention. *F_ST_* did not show an isolation by distance pattern, while *D_est_* did. *D_est_* is a good measure of genetic differentiation among populations in its own terms but, it is not yet conclusive how it can be interpreted concerning evolution of sets of populations [62].

Clustering of individuals using STRUCTURE on the microsatellite dataset did not reveal genetic patterns among populations within the two areas. These results are in agreement with the *F_ST_* results of the mitochondrial DNA dataset. The overall low level of genetic differentiation of the mitochondrial markers, together with the occurrence of shared haplotypes among populations, and limited differences between haplotypes, suggests that no strong barrier to gene flow exists within the NTS and STS areas. For example, using mitochondrial markers Miller et al. [14] and Addamo et al. [63] did not observe genetic structuring in populations of *Desmophyllum dianthus* at similar spatial scales. However, Miller et al. [14] using mtC did find differences in sequences along a depth gradient, as observed by Costantini et al. [2]. In red coral the mtC marker seems to be sufficiently polymorphic for detecting genetic structuring at scales of hundreds kilometres. Therefore, mtC could be a suitable marker for use in phylogeographic studies at the Atlanto-Mediterranean scales, to clarify the evolutionary history of this species.

DAPC analysis of both mitochondrial and microsatellites data sets, showed a clear pattern of divergence between Northern Tyrrhenian and Southern Tyrrhenian Sea red coral populations. This pattern of genetic structuring shows the occurrence of a barrier to gene flow between STS and NTS, determined by geomorphology, hydrodynamics, habitat features, or by bio-ecological characteristic of the species. NTS and STS are separated by a putative biogeographical barrier dividing the Ligurian and Tyrrhenian Seas [64]. In addition, it should be considered that in summer, when the planula larvae are released, the Tyrrhenian circulation has a fragmented pattern with local gyres [65] that could limit the dispersal and mixing of larvae, thus acting as a barrier to gene flow. Moreover, events that occurred in the past may have contributed to the observed pattern (e,g. sea level oscillations prior to the last glacial maximum 20000 years ago).

### Conclusions

High genetic structuring observed in mesophotic *Corallium rubrum* populations, together with a lack of connectivity between shallow-water (above 50 m depth) and deep-water (below 50 m depth) populations [2], [23], suggests that it is unlikely that deep-water populations may act as refugia and/or larval supply for shallow-water populations or *vice versa*, thus enhancing the resilience of the species. Harvesting pressure, coupled with other anthropogenic and natural disturbances will likely increase fragmentation of mesophotic populations. This will result in an erosion of their population size, genetic diversity (i.e. evolutionary potential) and resilience; ultimately threatening the species with risks of local extinctions. Long term survival of these heavily exploited populations has to rely on local recruitment. Preservation of the reproductive potential, supported mainly by larger and more valuable colonies, has to be a priority for the conservative management of the species. Therefore, management of red coral harvesting in the mesophotic habitats should be defined at a regional (or sub regional) level.

The discovery that red coral in the mesophotic zone is genetically structured; together with the evidence of a barrier to gene flow suggest the need to protect these vulnerable marine ecosystems by creating deep-sea marine protected areas. Establishment of marine protected areas along the continental shelf, inclusive of the deep and off-shore reefs, can enhance preservation of commercially exploited, long-lived, sessile invertebrates [66]. Previous studies showed the effectiveness of marine reserves in increasing the maximum size of shallow red coral colonies and influencing a shift toward mature populations [4].

Future efforts should focus on characterising connectivity patterns and vulnerability of mesophotic red coral populations. We suggest that this occurs in the form of 1) mapping Mediterranean deep red coral populations and 2) using a multidisciplinary approach to study population structure and dynamics of these populations. Finally, extending genetic studies to other marine species in the mesophotic zone (e.g. black coral) is needed to characterise connectivity patterns and vulnerability of these ecosystems.
